# Optical Riblet Sensor: Beam Parameter Requirements for the Probing Laser Source

**DOI:** 10.3390/s16040458

**Published:** 2016-03-30

**Authors:** Juliane Tschentscher, Sven Hochheim, Hauke Brüning, Kai Brune, Kay-Michael Voit, Mirco Imlau

**Affiliations:** 1 Department of Physics, Osnabrueck University, Barbarastrasse 7, Osnabrueck 49076, Germany; shochheim@uos.de (S.H.); mirco.imlau@uni-osnabrueck.de (M.I.); 2 Fraunhofer Institute for Manufacturing Technology and Advanced Materials IFAM, Bremen 28359, Germany; hauke.bruening@ifam.fraunhofer.de (H.B.); kai.brune@ifam.fraunhofer.de (K.B.); 3 Caesar Research Center, Ludwig-Erhard-Allee 2, Bonn 53175, Germany; kay-michael.voit@caesar.de

**Keywords:** optical riblet sensor, wave fronts and ray tracing, drag reduction, laser beam parameters, riblet degradation

## Abstract

Beam parameters of a probing laser source in an optical riblet sensor are studied by considering the high demands on a sensors’ precision and reliability for the determination of deviations of the geometrical shape of a riblet. Mandatory requirements, such as minimum intensity and light polarization, are obtained by means of detailed inspection of the optical response of the riblet using ray and wave optics; the impact of wavelength is studied. Novel measures for analyzing the riblet shape without the necessity of a measurement with a reference sample are derived; reference values for an ideal riblet structure obtained with the optical riblet sensor are given. The application of a low-cost, frequency-doubled Nd:YVO${}_{4}$ laser pointer sufficient to serve as a reliable laser source in an appropriate optical riblet sensor is discussed.

## 1. Introduction

Nano- and microstructured grooves on turbulent boundary layers, so called *riblets*, potentially reduce skin friction on the order of 9.9% [1,2] and, thus, have a major impact on drag engineering of aircrafts in the context of kerosine consumption (≡ costs) and emission of greenhouse gases (for an overview, *cf.* [3,4,5]). Great demands are made on the geometrical shape of the grooves as deviations from theoretically designed (ideal) riblets of only a few percentage considerably reduce the drag-reducing functionality [6,7]. In the fields of riblet fabrication and maintenance, it is thus necessary to detect deviations of the geometrical shape with high reliability and high precision at high speed. Preference is given to a contact-free, optical sensor concept, such as those recently proposed in [8]: a laser beam incidences normal to the riblet layer and the intensity distribution of the scattered light is applied for the inspection of the riblet’s geometrical shape. Although the principle proof of the sensors’ functionality has been successfully demonstrated, nearly nothing is known about the requirements for the applied probing light source. In particular, there are no reports on the impact of wavelength, power, beam divergence, and light polarization on the precision and reliability of the sensor. Without this knowledge, beam parameters that are best suited and appropriate for the development of an optical riblet sensor to determine shape deviations remain unknown, but the risk of laser-induced damage of the riblets may also exist.

Nowadays, the process management for the fabrication of efficient riblets with high geometrical quality and reproducibility is state-of-the-art and realized either by means of laser-etching, lumbering, grinding or embossing (see [9,10,11,12,13,14,15,16]). Controlling the manufacturing process by direct and indirect laboratory-based imaging techniques, e.g., confocal or electron microscopy (*cf.* SEM mentioned in [11]), provides precise insight to the geometry of fabricated riblets such as its periodicity, structure height, shape and surface roughness. However, these methods are time-consuming or require pre-treated samples of given dimensions and, thus, are not considered in the development of contact-free riblet sensor concepts. Other methods, mainly developed for contact-free sensor technologies of nanostructures in silicon industries, are not appropriate for the inspection either of grooves that are characterized by angles between 20 and 60${}^{{}^{\circ}}$ or of topographies that obey height profiles of 50–100 µm. Because of the lack of specific riblet sensor concepts, Meyer *et al.* proposed to use diffraction of a probing light at the laterally periodically arranged riblets to inspect the riblet geometry [17] and show that this concept allows for the inspection of the riblet periodicity and duty cycle. For the purpose of determining geometrical differences between real and ideal riblets, a technical variant of this concept is presented in [8], based on scattering of an incoming coherent light wave at the riblets. Multiple reflections inside the riblet grooves yields light waves predominantly in directions orthogonally and under angles of ±45${}^{{}^{\circ}}$ with respect to the grating vector of the riblets. The characteristic feature of this concept is that the intensity of the scattered beams is directly dependent on the structure parameters of the riblets that can be measured at an appropriate distance (several centimeters) to the riblet layer within several milliseconds and, thus, is very well suited for the development of a high-sensitive, contact-free optical riblet sensor.

This paper deals with the important demands on a light source used to determine geometric changes of riblets with respect to a reference structure at high precision (≪10%) with the sensor concept of [8] as an example. We use riblets that have been fabricated by stamping according to the procedure described in [11]; geometric changes (on the order of 1%–50%) are generated by mechanical treatment of the as-fabricated riblets. Parameters under study are wavelength, beam divergence, laser power, and light polarization and are analyzed with respect to the sensors’ signal-to-noise-ratio (SNR). As a result, essential boundary requirements for a laser source are derived that ensure a SNR ≫10, including a beam divergence of at least ≈1 × 10${}^{-30}$, linear light polarization (1:1000) and a minimum power of 82 µW. It is shown that a high-precision, contact-free optical sensor can be designed by using a low-cost, frequency-doubled, linearly polarized laser pointer (wavelength 532 nm) with 1 mW power much below the laser-induced damage threshold of riblet lacquer; geometry changes of riblets with respect to a reference in the order of 1% are reliably detected.

## 2. Riblet Samples

**Figure 1:**
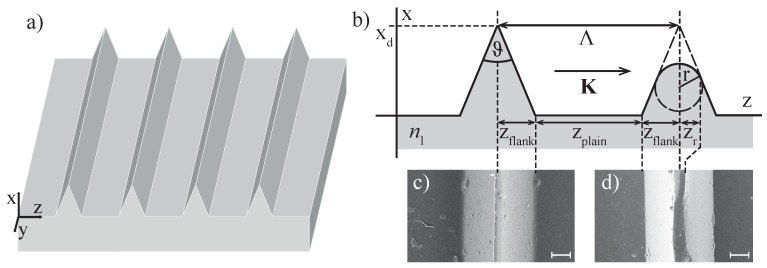
(**a**) schematic, three-dimensional representation of the riblet geometry under study. (**b**) (x,z)-cut through one of the riblet three-cornered stripes with ideal (left part) and deviated (right part) shape. K is the wave vector of the spatial periodic structure with K∥z-coordinate, |K|=2π/Λ and the period length Λ = 100 µm. Groove width $z_{\mathrm{plain}}\;\approx \;60$ µm, groove depth $x_{d}\;=\;$50 µm, width of the stripes $2z_{\mathrm{flank}}\;=\;40$ µm, and tip angle $\vartheta =45^{{}^{\circ}}$. The projection of the tip radius on the *z*-coordinate, $z_{r}$, can be used as striking measure for the deviation of the geometrical shape. The lacquer is characterized by its index of refraction $n_{l}$. (**c**) scanning electron microscopy (SEM) image of a riblet sample fabricated by stamping grooves into a lacquer. (**d**) SEM image of a riblet with deviated shape.

Our investigations were performed with riblet structures that are appropriate for drag-reduction. The samples were fabricated by stamping grooves into a lacquer as described elsewhere [11]. Figure 1a schematically shows a three-dimensional sketch of the underlying riblet geometry that is characterized by a spatial periodic sequence of three-cornered stripes.

The wave vector K of this surface relief grating is directed along the *z*-coordinate with |K|=2π/Λ and the period length Λ = 100 µm. The key measures describing the geometrical features are depicted in Figure 1b showing the (x,z)-cut through one of the riblet three-cornered stripes with ideal shape (left tip) with a groove width $z_{\mathrm{plain}}\;\approx \;60$ µm, a groove depth $x_{d}\;=\;$50 µm, a width of the stripes $2z_{\mathrm{flank}}$ = 40 µm, and a tip angle $\vartheta =45^{{}^{\circ}}$. An appropriate scanning electron microscope (SEM) image of a typical as-fabricated sample is shown in Figure 1c, and the scale is 10 µm. The high quality of the grooves is striking with only a few and widely scattered lacquer particles and/or droplets (below 1 µm; if required for high-sensitivity measurements, it is possible to remove these by wet cleaning procedures).

In contrast, the right part in Figure 1b shows a cut through a three-cornered stripe with deviated shape, particularly at its tip. A rounding of the tip—as shown—is very likely due to erosion during flight operation and is described using the tip radius *r*. A SEM image of a considerably treated sample with a severe tip damage is shown in Figure 1d. Here, the groove depth is reduced to $x_{d}\;=\;\left(40\pm 2\right)$ µm that corresponds with a structural deviation of 20% or a complete loss of the drag-reduction functionality. It becomes obvious, that the projection of the tip radius on the *z*-coordinate, $z_{r}$, can be used as a striking measure for this type of deviation of the geometrical shape. For the two SEM images, we find $2z_{r}\;=\;\left(2\pm 1\right)$ µm (as-fabricated sample) and $2z_{r}\;=\;\left(8\pm 2\right)$ µm (damaged sample).

The deviation of the riblet shape (Figure 1d) has been generated by mechanical treatment by means of a lathe. With this procedure, it was possible to fractional alter the tips, while keeping the groove width itself unaffected. For systematic studies, as shown below, a sample series with structural alterations in the range of 1%–50% was generated.

## 3. Riblet Sensor

The present study has been performed using the riblet sensor described in [8]. In this chapter, we focus on the important optical response of riblets that is the physical basis for the optical sensor concept and important for the evaluation of demands for the light source. We will further present the optical setup of the sensor itself and the respective measures allowing for the precise and reliable determination of deviations in the riblet shape. More details can be found in [8].

**Figure 2:**
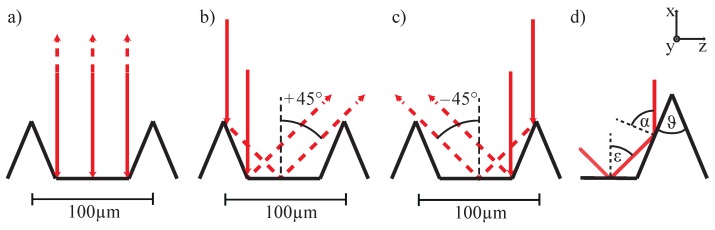
Ray tracing of the optical far-field response of a riblet structure for the case of exposure with a plane wave. (**a**) reflection at the plains of the grooves results in the appearance of a beam counterdirected (+x-axis) to the incoming beam; (**b**,**c**) part of the incident light hits the groove flanks and is reflected twice on the surface of the lacquer; and (**d**) the characteristic reflection angles at the groove flanks are $\alpha \;=\;67.5^{{}^{\circ}}$ and $\varepsilon \;=\;45^{{}^{\circ}}$ (measured normal to the surface).

The optical far-field response of a riblet structure for the case of exposure with a plane wave, homogeneous in intensity, is analyzed by means of ray-tracing in Figure 2.

**Figure 3:**
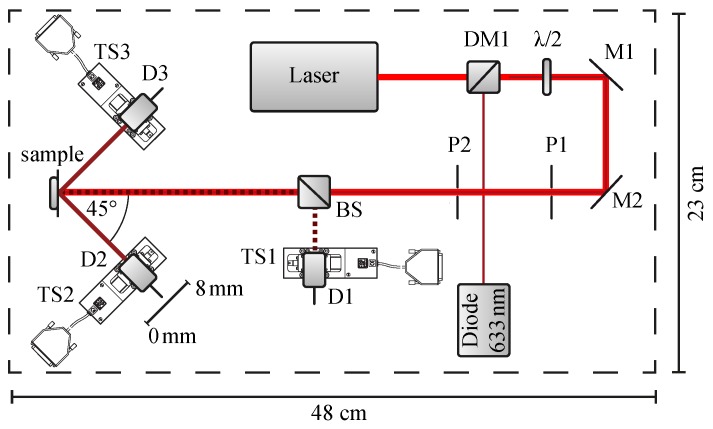
Scheme of the optical setup of the riblet sensor described in [8]. L1: laser source for probing, D1-3: SI-PIN-diodes, BS: beam-splitter, M1,2: mirrors, DM1: dichroic mirror, TS1-TS3: motorized translation stages, *λ*/2: wave retarder plate, L2: laser source at 633 nm for adjustment purposes. The distance *δ* between riblet sample and Si-PIN-diodes D1 is δ=287(2) mm, between riblet sample and D2, D3 δ=47(2) mm. For details, see text.

We here assume an ideal periodic structure and light incidence along the −x-coordinate, *i.e.*, normal to the wave vector K. For reasons of clarity, the ray-tracing results are distinguished for light reflected from the plains of the grooves (a), and the flanks (b,c). Light reflections from the plains of the grooves results in a counter-directed beam propagation in +x-direction (0 degrees with respect to the incoming light beam) with an intensity that is determined by the reflectivity of the lacquer. To the greatest extent, the lacquer is transparent over the visible and near-infrared spectral range; thus, the reflectivity is given by $R_{\mathrm{lacquer}}={\left(n{\left(\lambda \right)}_{l}-1\right)}^{2}/{\left(n{\left(\lambda \right)}_{l}+1\right)}^{2}$, with $n{\left(\lambda \right)}_{l}$ being the refractive index of the lacquer at wavelength *λ*. Due to a strong optical inhomogeneity of the lacquer layer itself, we neglect multiple internal reflections in the calculations of the intensity of the reflected beam. A fraction of the incident light hits the groove flanks. As it is demonstrated by the ray tracing result individually for the right and left flanks in Figure 2c,d, light is reflected twice in this case. According to the tip angle of $\vartheta =45^{{}^{\circ}}$, the angles of incidence for the first and second reflection are $\alpha \;=\;67.5^{{}^{\circ}}$ and $\varepsilon \;=\;45^{{}^{\circ}}$, respectively (see Figure 2d. As a characteristic feature of the riblet structure, the two-fold reflected beam is able to pass the nearest-neighboring flanks without losses and finally encloses an angle of $45^{{}^{\circ}}$ (and $-45^{{}^{\circ}}$) with the incoming beam. With these considerations, the far-field scattering pattern of the riblet structure is characterized by three striking features: one reflection in 0${}^{{}^{\circ}}$-and two in ±45${}^{{}^{\circ}}$-direction, all restricted to the plane determined by incoming and reflected beams and the direction of the riblet grating vector. Of major importance for the purpose of an optical sensor, it is noteworthy that the signals at 0${}^{{}^{\circ}}$ and ±45${}^{{}^{\circ}}$ contain unique information on the riblet geometry: the maximum around 0${}^{{}^{\circ}}$, originating solely from the plains of the grooves, can be used to measure the absolute value of the reflectivity of the lacquer and is not at all affected by any deviations in the structure of the three-cornered stripes. In contrast, the intensity distribution around ±45${}^{{}^{\circ}}$ is proportional to the lengths of the flanks that are responsible for the first reflection. In other words, a maximum intensity in $\pm 45^{{}^{\circ}}$-direction is present only for an ideal riblet structure and decreases linearly with decreasing groove depth, *i.e.*, increasing tip radius *r*. It is the task of the riblet optical sensor to precisely determine these three reflected intensities such that the degree of deviation from a theoretically ideal (or alternatively from a reference sample) is reliably determined with high precision as will be shown below. The experimental setup is shown schematically in Figure 3.

**Figure 4:**
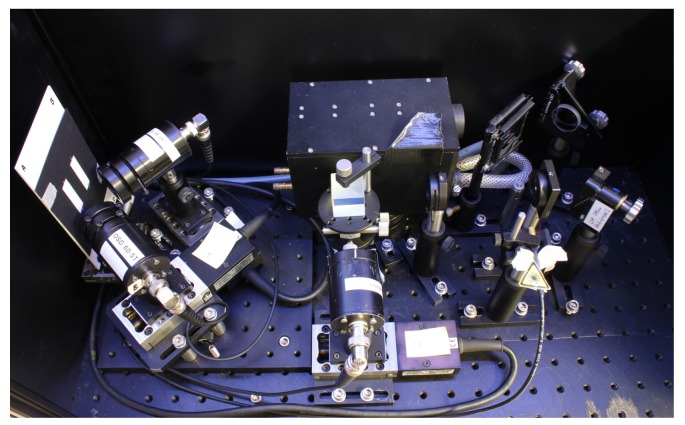
Photograph of the experimental setup as described above.

Light from a linearly polarized, unexpanded laser source is reflected by two mirrors such that it impinges normal to the sample surface. The direction of the light polarization with respect to the samples’ grating vector $K_{\mathrm{riblet}}$ is adjusted using a λ/2 wave retarder plate (the direction of $K_{\mathrm{riblet}}$ is in plane, as denoted in the figure); the diaphragms P1,2 reduce unwanted scattered light from the laser output. The far-field optical response of the riblet is detected in the directions of the main reflections by Si-PIN-diode D1 via beamsplitter BS for the counterdirected reflection from the plains of the grooves ($0^{{}^{\circ}}$-beam) and by D2,3 for the reflection from the riblet flanks ($\pm 45^{{}^{\circ}}$ beams). The diodes are equipped with diaphragms (d=1 mm) and are mounted on motorized linear stages TS1-3 (Newport Corporation, Irvine, CA, USA, model *LM07*, max. travel range: 8 mm, unidirectional repetition precision: 50 nm), thus it is possible to scan the scattering intensity distribution from 0–8 mm (*cf*. Figure 3) that corresponds with an angular range of $\approx \pm 5^{{}^{\circ}}$ (due to this measuring procedure, all data scans shown below are given as a function of spatial coordinate of the linear stage). The diameter of 1 mm is chosen such that the aperture acts as frequency filter in order to suppress fringe pattern due to diffraction phenomena [8]. A laser diode (λ=633 nm), coupled collinearly to the beam path via a dichroitic mirror DM1, is used for adjustment purposes, in case of probing wavelengths in the near-infrared spectral range. The measures of the optical setup, of which a photo is shown in Figure 4, are: 0.23×0.48×0.25 m${}^{3}$ (total weight about 10 kg).

Considering our optical setup, the optical response of the riblet is measured by scanning the reflected intensity within the plane of incidence yielding a scattering intensity profile as will be shown in the experimental part below. Using these data sets, it is possible to determine the signals $S^{0}$ and $S^{\pm 45}$ via integration:

(1) $$\begin{array}{cc} S^{0,\pm 45} & ={\left.\int_{0\;\text{mm}}^{8\;\text{mm}}I_{\text{deg}}^{0,\pm 45}\left(\zeta \right)d\zeta \right|}_{\text{shutter}\;\text{open}} \end{array}$$

In what follows, the impact of various beam parameters on the signal-to-noise ratio (SNR) will be studied. For this purpose, the reflected signals are measured also for the particular case of zero probing light for each sample, *i.e.*, with laser light off. These noise signals are given by:

(2) $$\begin{array}{cc} N^{0,\pm 45} & ={\left.\int_{0\;\text{mm}}^{8\;\text{mm}}I_{\text{deg}}^{0,\pm 45}\left(\zeta \right)d\zeta \right|}_{\text{shutter}\;\text{closed}} \end{array}$$

## 4. Riblet Sensor Measures without Reference Sample

According to the considerations made above, and as significant further development to the state-of-the-art knowledge presented in [8], it is possible to retrieve a set of important information on the geometrical features of the riblet structures as well as on the lacquer itself from the analysis of the scattering signals in 0${}^{{}^{\circ}}$, and $\pm 45^{{}^{\circ}}$-directions—even within a single measurement, *i.e.*, without the necessity of measuring a reference riblet sample. As a first measure, we here introduce the *plain efficiency* $\eta_{\mathrm{plain}}$ defined by the scattered ($0^{{}^{\circ}}$) power normalized to the incident power. To understand the relation between $\eta_{\mathrm{plain}}$ and the geometrical riblet parameters, we examine the fraction of incident light power $P_{0}$ that falls onto the plains. This fraction is equal to the ratio of plain width and period length $z_{\mathrm{plains}}/\Lambda$, so that the reflected scattering power in 0${}^{{}^{\circ}}$-direction can be expressed by $S^{0}\;=\;P_{0}R\left(0^{{}^{\circ}}\right)z_{\mathrm{plains}}/\Lambda$. Here, $R(0^{{}^{\circ}})$ denotes the reflectivity of the riblet lacquer at zero angle of incidence. The plain efficiency $\eta_{\mathrm{plain}}$ then is expressed by

(3) $$\eta_{\mathrm{plain}}=\frac{S^{0}}{P_{0}}=\frac{R\left(0^{{}^{\circ}}\right)z_{\mathrm{plains}}}{\Lambda }$$

It is obvious that $\eta_{\mathrm{plain}}$ can be applied for the determination of the lacquer reflectivity, $R\left(0^{{}^{\circ}}\right)\;=\;\eta_{\mathrm{plain}}\Lambda /z_{\mathrm{plains}}$, of as-fabricated riblets. However, it can be used also for the inspection of riblets with any type of degradation if related to structural deviations of the three-cornered stripes, *i.e.*, if $z_{\mathrm{plains}}$ and/or Λ remain unaffected. The knowledge of the lacquer reflectivity is important in the framework of flight maintenance as it reveals a degradation of the lacquer itself, e.g., induced by long-term exposure to ultraviolet light. A second measure of the optical sensor is the *flank efficiency* given by the scattering power $S^{\pm 45}$ in $\pm 45^{{}^{\circ}}$-direction normalized to the incoming laser power $P_{0}$:

(4) $$\eta_{\mathrm{flank}}^{\pm 45}=\frac{S^{\pm 45}}{P_{0}}=\frac{z_{\mathrm{flanks}}^{\pm 45}R\left(67.5^{{}^{\circ}}\right)R\left(45^{{}^{\circ}}\right)}{\Lambda }$$

In this case, the fraction of incident light exposing the left and right flanks is determined by the ratios $z_{\mathrm{flanks}}^{\pm 45}/\Lambda$ and—according to the two-fold reflection—the reflectivities: $R\left(67.5^{{}^{\circ}}\right)R\left(45^{{}^{\circ}}\right)$. The flank efficiency can be applied for the detection of tip degradations, as $\eta_{\mathrm{flank}}^{\pm 45}$ is a linear dependent on $z_{\mathrm{flank}}$ and therefore $\eta_{\mathrm{flank}}$ decreases linearly with any type of losses of the geometrical features of the stripes. In the limit of a plane lacquer without grooves, $\eta_{\mathrm{flank}}^{\pm 45}$ becomes zero. The riblet degradation often is described by the tip radius *r*. We therefore introduce $\left(z_{\mathrm{flanks}}^{\pm 45}-z_{r}\right)$ to describe the deviation with respect to the ideal structure because it relates with the tip radius via $r=z_{r}/\sin\left(67.5^{{}^{\circ}}\right)$. Using Equation (4), it is possible to determine *r* from the measurement of the flank efficiency via

(5) $$r=\left[z_{\mathrm{flanks}}^{\pm 45}-\frac{\eta_{\mathrm{flank}}^{\pm 45}\left(z_{r}\right)\Lambda }{R\left(67.5^{{}^{\circ}}\right)R\left(45^{{}^{\circ}}\right)}\right]\frac{1}{\sin(67.5^{{}^{\circ}})}$$

A third measure of the optical sensor is the *quality efficiency*, which is not dependent on the measurement of the incident laser power

(6) $$\eta_{\mathrm{quality}}^{45}=\frac{S^{\pm 45}}{S^{\pm 45}+S^{0}}=\frac{z_{\mathrm{flanks}}^{\pm 45}R\left(67.5^{{}^{\circ}}\right)R\left(45^{{}^{\circ}}\right)}{z_{\mathrm{flanks}}^{\pm 45}R\left(67.5^{{}^{\circ}}\right)R\left(45^{{}^{\circ}}\right)+R\left(0^{{}^{\circ}}\right)z_{\mathrm{plains}}}=\frac{c}{c+\frac{z_{\mathrm{plains}}}{z_{\mathrm{flanks}}}}$$

with the ratio of lacquer reflectivities $c=R\left(67.5^{{}^{\circ}}\right)\cdot R\left(45^{{}^{\circ}}\right)/R\left(0^{{}^{\circ}}\right)$.

Besides ray optics, it is necessary to discuss the (possible) impact of diffraction of the incoming light at the periodic structure of the riblet. We assume a beam diameter much larger than the period length Λ and a high quality of the riblet periodicity at least within the exposed area. Then, the appearance of beams of order *m* diffracted from the riblet structure are described using the grating equation: Λ=mλ/sinθ with the angle *θ* measured between incident and diffracted beams and the wavelength *λ*. Considering a laser beam with beam divergence $\theta_{\mathrm{bd}}$, we can express the spatial separation between zero and first order at a given distance *δ* by:

(7) $$\begin{array}{cc} d(\lambda ,\theta_{\mathrm{bd}})\; & =\;\delta \cdot \tan(\theta )\\ & =\;\delta \cdot \tan\left[\sin^{-1}\left(\frac{\lambda }{\Lambda }-\sin\left(\theta_{\mathrm{bd}}\right)\right)\right] \end{array}$$

From the experimental data at real structures shown below, it becomes obvious, that light diffraction superimposes the reflected signals and can be seen clearly in the detected scattering pattern in $0^{{}^{\circ}}$ direction. At the same time, the diffraction pattern is not resolved in $\pm 45^{{}^{\circ}}$-direction. We note, that—according to Equations (1) and (2)—the diffraction pattern is not analyzed as it follows from the integral definition of our measures.

## 5. Experimental Section: Impact of Beam Parameters on SNR

### 5.1. Laser Power

**Figure 5:**
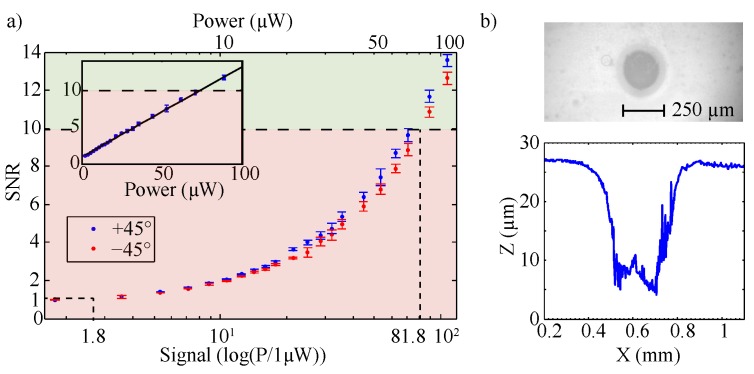
(**a**) signal-to-noise-ratio related to the quality efficiency as a function of laser power in a semi-logarithmic plot. A noise value of 1.8 µW is obtained (SNR equal to unity); the threshold power for a precise determination of the quality efficiency is 81.8 µW (SNR = 10). The insert shows the same data set within a linear-linear plot. (**b**) damage in the riblet lacquer caused at laser induced damage threshold intensity of (250±5) MW/m${}^{2}$.

This subsection deals with determination of the threshold values of the laser power that allow for a precise measurement of $\eta_{\mathrm{quality}}$ at least with a limiting SNR >10 but does not result in a laser-induced damage of the riblet lacquer. For this purpose, a YVO${}_{4}$:Nd laser (λ=532 nm) was applied as probing light source and the power was varied from 1 µW to 100 µW using a combination of wave retarder plate and Glan–Thompson polarization prism in the beam path (*cf.* Figure 3). Figure 5a shows the obtained values of the signal-to-noise-ratio (SNR) for signals in $\pm 45^{{}^{\circ}}$ direction as a function of laser power on a semi-logarithmic scale (the inset of Figure 5a shows the same data in a linear-linear plot).

The measurement was performed under ambient daylight conditions (10,000 lux) equal to a noise level of about 1 µW (SNR = 1). A linear increase of the SNR with increasing laser power is obvious; an SNR ≥10 is reached at a threshold value of the laser power of (81.8±0.1) µW = 1 marked by the dashed line. In a second series, the laser intensity has been increased up to 10 GW/m${}^{2}$ to determine the laser induced damage threshold (LIDT) value, *i.e.*, the intensity that results in a thermo-optical, irreversible damage of the riblet lacquer. For this purpose, a focal lens with f=100mm was placed within the beam path. For each intensity, the lacquer was exposed for a duration of 1 s (the typical measurement time of the riblet optical sensor) and the exposed surface area was inspected for damages using a microscope and a low-coherence interferometer. Figure 5b shows a photograph of a typical laser-induced damage and its topography, here obtained at an intensity of (250±5) MW/m${}^{2}$. The depth of the damage of 20 µm exceeds the groove depth to a large extent; its diameter (250 µm) resembles the beam waist of the incoming laser beam. The measurements were repeated 10 times for each intensity. As a result, an LIDT value of (225±10) MW/m${}^{2}$ was obtained.

### 5.2. Wavelength and Beam Divergence

In this subsection, we study the impact of wavelength *λ* and beam divergence $\theta_{\mathrm{bd}}$ on the optical response using the laser systems listed in Table 1 and the distance $d(\lambda ,\theta_{\mathrm{bd}})$ between main and side peaks of the 0${}^{{}^{\circ}}$ scattering pattern. For the purpose of our measurements, the setup was operated with different laser systems with wavelengths ranging from 405 nm up to 785 nm. Table 1 summarizes also the further parameters (power, beam divergence, and laser safety class); all lasers are linearly polarized and are commercially available (between 100-EUR and 40,000-EUR).

The beam divergence has been measured by means of distant-dependent determination of beam intensity profiles.

**Figure 6:**
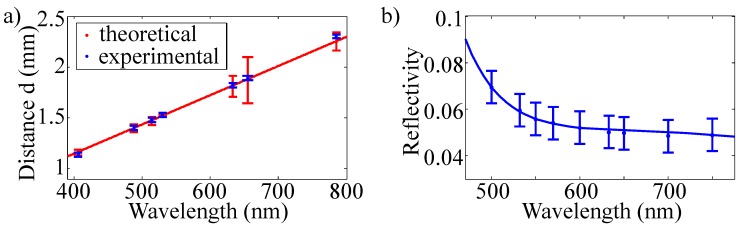
(**a**) wavelength dependence of the distance $d(\lambda ,\theta_{\mathrm{bd}})$ between main and side peaks of the 0${}^{{}^{\circ}}$ scattering pattern (as marked below in Figure 9a) measured at identical spots of an as-fabricated riblet sample and using the laser systems listed in Table 1. The red line denotes a fit of function Equation (7) to the data points (blue). (**b**) wavelength dependence of the reflectivity; blue line: guide to the eyes.

Figure 6 shows the result of our measurement obtained at an identical spot of an as-fabricated riblet sample.

A (nearly) linear increase of *d* with increasing wavelength is obvious. According to Section 3, we here assume that the three distinct scattering features in the $0^{{}^{\circ}}$-pattern represent zero (main peak) and first order (side peaks) diffracted beams from the laterally periodic plains of the riblet, *i.e.*, from a reflection grating with period length Λ=100 µm. Thus, the scattering angle *θ* between the beams depends on the wavelength according to Equation (7). A fit of Equation (7) to the experimental data is plotted in Figure 6a (red line); the coincidence between experimental data points and fitting function is striking. We note that an impact of the beam divergence on the scattering pattern is not found within the experimental conditions of the sensor setup; particularly for the wavelength λ=488 nm, two laser systems with $\theta_{\mathrm{bd}}=4.65\left(39\right)\times 10^{-4}$ (frequency-doubled OPSL) and $\theta_{\mathrm{bd}}=5.82\left(49\right)\times 10^{-4}$ (Ar${}^{+}$-ion laser), *i.e.*, a difference of $\theta_{\mathrm{bd}}$ of about 25%, revealed both an experimentally determined value of d=1.40(2) mm.

The wavelength further affects the SNR related with the flank, plain and quality efficiencies by means of the dispersion of the reflectivity R(λ). We note, however, that the impact of R(λ) on the SNR can be neglected for an SNR ≫10.

### 5.3. Polarization

**Figure 7:**
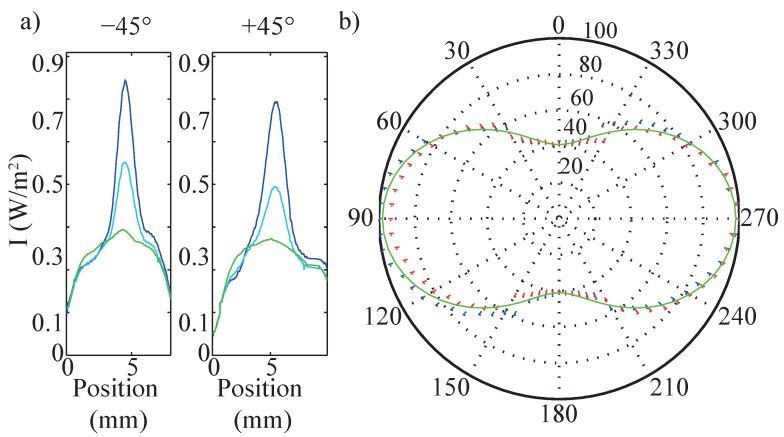
(**a**) intensity distribution at 0${}^{{}^{\circ}}$- (perpendicular to the lattice vector), 45${}^{{}^{\circ}}$- and 90${}^{{}^{\circ}}$- (parallel to the lattice vector) polarized light. (**b**) polar diagram of the measured intensities at $\pm 45^{{}^{\circ}}$ at various angles *δ* between e and K. The intensity values are normalized to the intensity maximum at *δ* = 90${}^{{}^{\circ}}$.

According to the ray tracing results and the definition of flank, plain and quality efficiency, the direction of the light polarization of the incident probing light with respect to $K_{\mathrm{riblet}}$ is of major importance for the strength of the scattering signals. The main reason is the pronounced dependency of the reflectivity on the angle of incidence, and, furthermore, the different behavior for parallel and perpendicular light polarization (*π*- and *σ*-polarized light). Figure 7a shows the impact of light polarization on the signal strength that has been measured for $e∥K_{\mathrm{riblet}}$ (green, *π*-polarized), $e⊥K_{\mathrm{riblet}}$ (dark blue, *σ*-polarized), and an angle of $\delta =45^{{}^{\circ}}$ between e and $K_{\mathrm{riblet}}$ (light blue), exemplarily for λ=488 nm and in $\pm 45^{{}^{\circ}}$-direction.

Qualitatively, the scattering patterns are comparable with each other and the patterns depicted below in Figure 9b,c, that all show a main scattering feature with a broad tail extending for increasing scan positions. However, the signal strength is strongly dependent on the angle *δ* between e and K; it is maximal for *σ*-polarized ($\delta =90^{{}^{\circ}}$) and minimal for *π*-polarized ($\delta =0^{{}^{\circ}}$) light while the $\delta =45^{{}^{\circ}}$ signal is measured having a signal amplitude in between. On the contrary, the scattering pattern in 0${}^{{}^{\circ}}$-direction is not affected by variation of *δ* (not shown). This behavior is studied in more detail for the $\pm 45^{{}^{\circ}}$-scattering pattern in the regime $0<\delta <360^{{}^{\circ}}$ in steps of 5${}^{{}^{\circ}}$, as shown in the polar plot of Figure 7b. The intensity values are normalized to the intensity at *δ* = 90${}^{{}^{\circ}}$ polarized light and vary between 40% (*π*-polarized) and 100% (*σ*-polarized).

**Figure 8:**
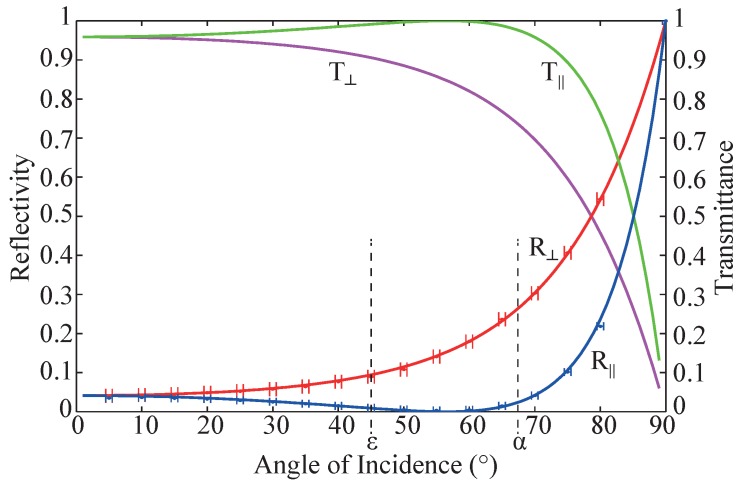
Reflectivity *R* and Transmittance *T* at an unstructured sample with perpendicularly (⊥) and parallel (||) polarized light plotted against angle of incidence. The refraction index of the riblet coating is *n*${}_{2}\;=\;$1.51. The significant angles of incidence at the riblet structure *α* and *ε* (*cf.* Figure 2d) are marked.

This pronounced dependency on the light polarization can be modeled using absolute values of the reflectivities related to the two particular angles of incidence α,ε (*cf.* Figure 2d) and *σ*- and *π*-polarized light. For this purpose, the reflectivity of the riblet lacquer is measured at different angles of incidence for both perpendicularly and parallel polarized light. The results are presented in Figure 8.

The solid lines show the Fresnel’s Equations plotted against angle of incidence for reflected and transmitted light at a surface with a refractive index of *n*${}_{2}\;=\;$1.51. The measured data is in good agreement with the theoretical curve. The marked angles *α* and *ε* are the characteristic angles of incidence of the riblet structure (*cf.* Figure 2d). Here, the reflectivity of perpendicularly and parallel polarized light differs significantly.

Using the angular dependency of the reflectivity and the geometrical considerations of Figure 2d, it is possible to model the dependency of the reflected intensity as a function of angle *δ* for *σ*- and *π*-polarized light. The modeling result is depicted as the green line in Figure 7b and shows excellent agreement with the experimental parameters.

From these investigations, it becomes clear, that deviations of the light polarization have a major impact on the SNR. For instance, using natural light polarization reduces the SNR by 35% and even a slight tolerance of $\delta \;=\;10^{{}^{\circ}}$ implies a riblet deviation of ≈5% as wrong interpretation of the sensors’ signal.

### 5.4. Data Scans

**Figure 9:**
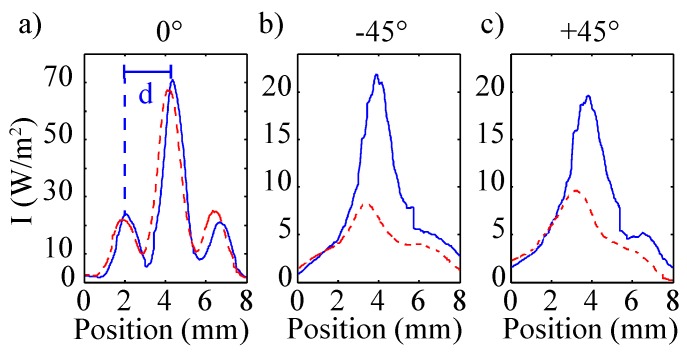
Scattered intensity patterns as a function of position. (**a**) shows the intensity around 0${}^{{}^{\circ}}$ detected with D1 (*cf.* Figure 3), (**b**,**c**) present the intensity around −45${}^{{}^{\circ}}$ and +45${}^{{}^{\circ}}$, respectively, measured with D2 and D3 (*cf.* Figure 3). The solid line shows the measurement of a non-degraded riblet structure as shown in Figure 1c, the dashed line is the result of the measurement of the degraded riblet structure shown in Figure 1d. The distance *d* between main peak and peak of first order is marked exemplarily for the non-degraded riblet structure in (**a**).

Typical data scans obtained with the optical sensor described above are shown in Figure 9, exemplarily for a frequency doubled Nd:YVO${}_{4}$ laser system (λ=532 nm, incident power: 150 µW).

The three plots correspond with the signals determined using Si-PIN-diodes D1 (0${}^{{}^{\circ}}$, Figure 9a), D2 (−45${}^{{}^{\circ}}$, Figure 9b), and D3 (+45${}^{{}^{\circ}}$, Figure 9c). Two data sets are shown in each plot: for an as-fabricated (solid) and mechanically pre-treated (dashed) riblet sample; the scattered intensity is plotted as a function of scan position. At 0${}^{{}^{\circ}}$, both plots reveal three scattering peaks: one main peak and two side peaks symmetrically positioned at distance *d* to the main peak. The distributions at $\pm 45^{{}^{\circ}}$ are mirror symmetric to each other; they feature a main peak with a broad tail extending for increasing scan positions (corresponds with larger scattering angles with respect to the incoming beam). The difference between the two samples, *i.e.*, between grooves of different depth, is striking: strong losses of more than 70% are obvious for the mechanically treated sample in the $\pm 45^{{}^{\circ}}$ plots. At the same time, the $0^{{}^{\circ}}$ plots remain nearly unaffected by the severe damage of the riblet tip. This observation is reasonable taking into account the ray tracing considerations of Section 3: the reflected power at $\pm 45^{{}^{\circ}}$ is directly proportional to the areas of the riblet flanks, while the $0^{{}^{\circ}}$ signal originates from reflection at the tails of the grooves. The three plots correspond with the signals determined using Si-PIN-diodes D1 (0${}^{{}^{\circ}}$, Figure 9a), D2 (−45${}^{{}^{\circ}}$, Figure 9b), and D3 (+45${}^{{}^{\circ}}$, Figure 9c). Two data sets are shown in each plot: for an as-fabricated (solid) and mechanically pre-treated (dashed) riblet sample; the scattered intensity is plotted as a function of scan position. At 0${}^{{}^{\circ}}$, both plots reveal three scattering peaks: one main peak and two side peaks symmetrically positioned at distance *d* to the main peak. The distributions at $\pm 45^{{}^{\circ}}$ are mirror symmetric to each other; they feature a main peak with a broad tail extending for increasing scan positions (corresponds with larger scattering angles with respect to the incoming beam). The difference between the two samples, *i.e.*, between grooves of different depth, is striking: strong losses of more than 70% are obvious for the mechanically treated sample in the $\pm 45^{{}^{\circ}}$ plots. At the same time, the $0^{{}^{\circ}}$ plots remain nearly unaffected by the severe damage of the riblet tip. This observation is reasonable taking into account the ray tracing considerations of Section 3: the reflected power at $\pm 45^{{}^{\circ}}$ is directly proportional to the areas of the riblet flanks, while the $0^{{}^{\circ}}$ signal originates from reflection at the tails of the grooves. The $0^{{}^{\circ}}$ signal is reduced only, if the reflectivity of the lacquer changes, e.g., by coating the samples with carbon (data not shown). Thus, the above considerations of the optical response of riblets are verified by experimental means, and it is possible to use the loss of the $\pm 45^{{}^{\circ}}$ and $0^{{}^{\circ}}$ signals as a measure for the groove depth and for reference, respectively.

## 6. Discussion

Section 4 reveals that theoretical (reference) values for an ideal riblet structure can be calculated and used for reference purposes, provided the knowledge of reflectivities at given angles of incidence. Using the results of Section 5.3, we can calculate these reference values for the given riblet structure. The results are given in Table 2 with the frequency-doubled OPSL-laser as an example. The values are obtained using Equations (3), (4) and (6) from Section 4: z${}_{\text{flanks}}/\Lambda$, z${}_{\text{plains}}/\Lambda$, $\eta_{\text{flank}}$, $\eta_{\text{plain}}$ and $\eta_{\text{quality}}$. The parameters applied for the determination of these results are also shown in the table: the values of riblet periodicity Λ and riblet height *h* as introduced in Figure 1, the reflection angles *α* and *ε* as shown in Figure 2 and the characteristic angle-dependent reflectivity values R(0${}^{{}^{\circ}}$), R(45${}^{{}^{\circ}}$) and R(67.5${}^{{}^{\circ}}$) as presented in Figure 8.

We like to note that these reference values can be used for calibration purposes of the riblet optical sensor. Particularly, they are very useful for setting threshold values that mark the degree of the structural breakdown of the riblets, e.g., in the framework of maintenance.

In general, and based on our findings, we can make a recommendation for the type of laser best applied in a riblet optical sensor: considering the laser wavelength, lasers with small wavelengths should be preferred since the reflectivity in $\pm 45^{{}^{\circ}}$-direction increases as a result of dispersion. However, we discovered strong losses in the reflectivity for wavelengths below 450 nm that are attributed to strong light scattering in the riblet lacquer itself. This particularly hindered the determination of the reflectivity for the 405 nm laser source, which thus should not be used for a sensor device. Using wavelengths in the visible spectral range further are advantageous from the viewpoint of a simple adjustment of the probing laser beam to a particular measurement point on the riblet surface; thus all laser systems with wavelengths below 700 nm can be taken into account. In addition, we need to consider the required laser power. Here, our study shows that the SNR improves with increasing power; however, the high-power lasers used here (the frequency-doubled OPSL and Ar${}^{+}$-ion laser) are the most expensive ones, feature the largest dimensions and are highly questionable in respect of laser safety issues—although neither of the lasers reached the LIDT of the riblet lacquer. As a result, it turns out that the low-cost (<100 EUR), frequency-doubled Nd:YVO${}_{4}$ laser pointer is sufficient to serve as a reliable laser source in an appropriate optical riblet sensor and also fulfills the demands on light polarization.

## 7. Conclusions

Our investigations show that it is extremely advisable to precisely study the impact of beam parameters of the laser source within an optical riblet sensor on the precision and reliability of riblet inspection. In particular, we find that the signal-to-noise ratio (SNR) can be affected very negatively by a weak intensity of the probing laser and/or by a low degree of light polarization polarization. Furthermore, it is necessary to align the direction of light polarization parallel to the geometrical features of the riblet. At the same time, beam divergence and laser wavelength play only a minor role in the optimization of the SNR, and the probing intensity is limited to maximum values according to the laser-induced damage features of the riblet lacquer. It is noteworthy that these results could only be obtained by a detailed inspection of the optical response of the riblet from ray and wave optics and allowed to derive novel measures for the optical riblet sensor. A severe advantage over the state-of-the-knowledge measure (*cf.* [8]) is the independency on a measurement with a reference sample; instead, theoretical values for an ideal riblet structure can be calculated and used for reference purposes. This is of particular advantage for measurement processes in the field. From the viewpoint of engineering an optical riblet sensor, it can be concluded that all demands for the development of a precise and reliable sensor can be fulfilled by choosing a low-cost (<100 EUR), frequency-doubled laser pointer (λ=532 nm).

## Figures and Tables

**Table 1:** Lasers systems used as probe lasers (see optical setup) and their characteristic parameters: power, wavelength, divergence, and laser safety class (classification according to IEC 60825-1 (2014); OPSL: optically pumped semiconductor laser). All laser systems feature linearly polarized light.

Laser System	Power (mW)	Wavelength (nm)	Beam Divergence (rad)	Laser Safety Class
InGaN laser diode	60	406	5.21(44)$\cdot 10^{-4}$	3B
frequency-doubled OPSL	2000	488	4.65(39)$\cdot 10^{-4}$	4
Ar${}^{+}$-ion laser	1400	488	5.82(49)$\cdot 10^{-4}$	4
Ar${}^{+}$-ion laser	2000	514	6.66(54)$\cdot 10^{-4}$	4
frequency doubled Nd:YVO${}_{4}$	1	530	5.26(29)$\cdot 10^{-4}$	2
He-Ne laser	3	632.8	1.40(23)$\cdot 10^{-3}$	3B
AlGaInP laser diode	5	655	1.00(69)$\cdot 10^{-3}$	3B
GaAs laser diode	40	785	1.20(19)$\cdot 10^{-3}$	3B

**Table 2:** Theoretical values.

Parameter	Value
riblet periodicity Λ	100 µm
riblet height h	50 µm
reflection angle *α*	67.5${}^{{}^{\circ}}$
reflection angle *ε*	45${}^{{}^{\circ}}$
reflectivity R(0${}^{{}^{\circ}}$)	0.041
reflectivity R(45${}^{{}^{\circ}}$)	0.094
reflectivity R(67.5${}^{{}^{\circ}}$)	0.265
z${}_{\text{flanks}}/\Lambda$	0.21
z${}_{\text{plains}}/\Lambda$	0.59
$\eta_{\text{flank}}$	$5\times 10^{-3}$
$\eta_{\text{plain}}$	$24\times 10^{-3}$
$\eta_{\text{quality}}$	0.1778

## References

[1] Bechert D.W., Bruse M., Hage W., van der Hoeven J.G.T., Hoppe G. (1997). Experiments on drag-reducing surfaces and their optimization with an adjustable geometry. J. Fluid Mech..

[2] Bruse M., Bechert D., van der Hoeven J.T., Hage W., Hoppe G. Experiments with conventional and with novel adjustable drag-reducing surfaces. Proceedings of the International Conference on Near-Wall Turbulent Flows.

[3] Walsh M. (1990). Effect of Detailed Surface Geometry on Riblet Drag Reduction Performance. J. Aircr..

[4] Bechert D.W., Bruse M., Hage W., Meyer R. (2000). Fluid Mechanics of Biological Surfaces and their Technological Application. Naturwissenschaften.

[5] Dean B., Bhushan B. (2010). Shark-skin surfaces for fluid-drag reduction in turbulent flow: A review. Philos. Trans. Royal Soc. Lond. A Math. Phys. Eng. Sci..

[6] Lee S.J., Lee S.H. (2001). Flow field analysis of a turbulent boundary layer over a riblet surface. Exp. Fluids.

[7] García-Mayoral R., Jiménez J. (2011). Drag reduction by riblets. Philos. Trans. Royal Soc. Lond. A Math. Phys. Eng. Sci..

[8] Imlau M., Bruening H., Voit K.M., Tschentscher J., Dieckmann V. Riblet Sensor-Light Scattering on Micro Structured Surface Coatings. http://arxiv.org/abs/1601.04694.

[9] Bixler G.D., Bhushan B. (2013). Bioinspired micro/nanostructured surfaces for oil drag reduction in closed channel flow. Soft Matter.

[10] Bixler G.D., Bhushan B. (2013). Fluid Drag Reduction with Shark-Skin Riblet Inspired Microstructured Surfaces. Adv. Funct. Mater..

[11] Stenzel V., Wilke Y., Hage W. (2011). Drag-reducing paints for the reduction of fuel consumption in aviation and shipping. Prog. Org. Coat..

[12] Denkena B., de Leon L., Wang B. (2009). Grinding of microstructured functional surfaces: A novel strategy for dressing of microprofiles. Prod. Eng..

[13] Han X., Zhang D. (2008). Study on the micro-replication of shark skin. Sci. Chin. Series E Technol. Sci..

[14] Hirt G., Thome M. (2007). Large area rolling of functional metallic micro structures. Prod. Eng..

[15] Klocke F., Feldhaus B., Mader S. (2007). Development of an incremental rolling process for the production of defined riblet surface structures. Prod. Eng..

[16] Marentic F., Morris T. (1992). Drag Reduction Article. U.S. Patent.

[17] Meyer U., Markus S., Dieckhoff S. (2014). Device for Testing the Quality of Microstructurization. U.S. Patent.

